# Neurocognitive Indices of Long COVID: Dysregulated Engagement of Inhibitory Control and Oscillatory Slowing

**DOI:** 10.3390/neurosci7050105

**Published:** 2026-09-26

**Authors:** David R. White, Katie S. Parker, Ksenija Marinkovic

**Affiliations:** Spatio-Temporal Brain Imaging Lab, Department of Psychology, San Diego State University, San Diego, CA 92182, USA; dwhite4@sdsu.edu (D.R.W.); katie.siu.parker@gmail.com (K.S.P.)

**Keywords:** PASC, Long COVID, electroencephalography, inhibitory control, error processing, neural synchrony, event-related theta, Go/NoGo task, oscillatory slowing, neuroinflammation

## Abstract

Persistent neurocognitive symptoms, such as “brain fog”, insomnia, or fatigue, are prevalent in PASC (post-acute sequelae of COVID-19, “Long COVID”), even after mild acute illness. However, evidence for the neural underpinnings of cognitive dysregulation and altered spontaneous neural activity is inadequate. EEG data were acquired during an inhibitory Go/NoGo task and wakeful rest from young, otherwise healthy adults with PASC approximately six months after recovery from mild COVID-19. Neurobehavioral indices were computed for correct and erroneous trials, resting state, and the questionnaire battery, and compared with a matched control (CONT) group of individuals not exposed to COVID-19. The PASC group reported higher cognitive difficulties, anxiety, depression, and sleep problems, consistent with the known symptomatic pattern. Despite intact task performance, neural indices indicated altered engagement of cognitive control in the PASC group on both correct and error trials, which was associated with less efficient responding. Moreover, co-oscillatory synchrony trended toward dysregulation during error commission in the PASC group. Altered neural indices of inhibitory control marginally correlated with spontaneous oscillatory slowing, potentially suggesting shared basic mechanisms. The overall pattern of PASC symptoms, altered neural activity during inhibitory control, and oscillatory slowing, is indicative of the core neural dysfunction characterizing PASC.

## 1. Introduction

Coronavirus disease (COVID-19) is associated with neurofunctional deficits with a varying symptom profile and severity, which can persist for months in a notable proportion of people after recovery from even mild acute infection [1,2,3,4,5,6,7,8,9,10]. Post-acute sequelae of SARS-CoV-2 infection (PASC), also termed “long COVID” [5], is usually diagnosed based on medical history and a chronic presentation of symptoms lasting at least one [11,12], or commonly two months or longer after acute illness [13]. Neuropsychiatric manifestations represent a significant portion of PASC symptoms, including cognitive difficulties with attention or memory, insomnia, fatigue, anxiety, and depression [5,6,7,8,9,14]. “Brain fog,” in particular, is a colloquial term for the inability to focus and concentrate, encompassing other aspects of dyscognition and “mental fatigue” [15,16,17]. Complaints of cognitive dysfunction are highly represented even after mild acute COVID-19 infection [7,9,15,18,19,20,21,22,23,24] and can persist for years after acute illness [3]. Although impaired cognition is a prominent PASC symptom, these findings primarily come from large reviews of self-reported data commonly obtained in clinical settings. While such descriptive insights are valuable, task-based evidence of their neural underpinnings—obtained using task-based paradigms in experimental settings—remains scarce. Progress is impeded by an inadequate understanding of the underlying neural mechanisms and the scarcity of objective measures. This is especially relevant since objective and subjective indices of cognitive dysfunction are not reliably correlated. For instance, people with PASC report experiencing “brain fog” even when their scores on standardized cognitive measures are not deficient [24]. Nonetheless, PASC symptoms can exert a significant burden on people living with PASC. Large scoping reviews indicate that executive functions are particularly affected [25,26,27,28]. Among them, response inhibition deficits are exceptionally long lasting, in contrast to other cognitive functions that improve over time [29].

Inhibitory control of behavior is a key dimension of the frontally mediated functions that underlie response suppression and execution in the service of flexible and efficient goal-directed behavior [30,31]. Commonly used inhibitory variants of a Go/NoGo task create a prepotency to respond on frequent Go trials, which needs to be deliberately suppressed on randomly interspersed, infrequent NoGo trials [32,33,34]. This feature makes it possible to investigate the neural underpinnings of correctly inhibited, as well as erroneous button-press responses that reflect error monitoring and post-error adjustments [33,35,36,37,38], which are fundamental to executive functions and behavior optimization [39,40].

Electroencephalography (EEG) is sensitive to changes in neurotransmission [41], making it well suited for studying the neural basis of PASC. EEG directly reflects oscillatory neural activity during task performance and can be analyzed within different frequency bands to characterize neural engagement [42,43]. Extensive evidence from our group and other studies indicates that frontocentrally mediated task-based event-related theta (er-theta) oscillations (4–7 Hz) are an index of cognitive engagement, including inhibitory control and post-error adjustments [34,36,44,45,46,47,48], as well as long-range synchrony (phase locking) [49,50]. However, evidence of the impact of PASC on the neural basis of task-based inhibitory control and error-related activity is lacking. Furthermore, at a more basic level, mechanistic insights into the neural underpinnings of PASC-related neural dysregulation of cell signaling are needed.

PASC is a multiorgan illness affecting different functional systems [51,52]. Proposed etiological factors encompass an array of pathophysiologic contributions, including chronic inflammation and immunological deficits, hypoxia, vascular and coagulation dysfunction, among others [5,9,11,15,53]. The impact of PASC on neural functions is of particular interest, given the prevalence of neurocognitive symptoms as reviewed above. EEG can provide insights into underlying neural dysregulation at a basic level as it directly reflects postsynaptic currents resulting in cell signaling. EEG signals are generated by the cerebral cortex due to the favorable biophysical properties of large neural ensembles and reflect the summation of excitatory and inhibitory currents [41]. Pyramidal cells—which represent most cortical neurons—are glutamatergic, excitatory projection neurons [54]. They form extensive neural networks through long-range oscillatory synchronization [55,56]. The remaining cortical neurons are GABA-expressing inhibitory interneurons. They are essential for stable neural network organization because they modulate excitation, gain, timing, and other properties of synaptic excitation [56,57,58,59,60]. The brain is a dynamic, interactive neural system [61] that relies on optimal neural balance, which underlies local synaptic activity and long-range neural communication, supporting healthy cognition [42,58,62,63,64]. In contrast, neural dysregulation due to network imbalance contributes to neurocognitive dysfunction [64,65,66]. Although PASC is multifactorial, persistent neuroinflammation has been proposed as a hallmark of neurocognitive symptoms [9,26,53,64,67,68]. Importantly, it impacts neurotransmission and is reflected in neural hyperexcitability [69,70,71]. Indeed, our proton magnetic resonance spectroscopy (1H-MRS) imaging study confirmed that the levels of GABA, the principal inhibitory neurotransmitter, are lower in people with PASC. Lower GABA mediated the impact of PASC on depression and was associated with poor sleep quality [72]. Other studies have documented that proinflammatory processes play an important role in depression, anxiety, and insomnia [73,74,75], which are comorbid with PASC. The current study was designed to provide complementary insight into possible neural dysregulation in people with PASC. Based on these lines of evidence, we hypothesize that resting-state EEG signals will demonstrate oscillatory slowing, suggestive of neural dysregulation. Extant EEG studies have reported a shift in oscillatory activity toward lower frequencies as part of the PASC clinical picture [76,77,78,79].

Overall, the available EEG evidence on PASC-related alterations in neural activity is scant, particularly regarding inhibitory control. It is often limited to studies conducted in clinical settings and in people hospitalized with either COVID-19 or PASC, neglecting cases of PASC arising from mild COVID-19. To address these gaps, the current study examined neurophysiological functions in young, previously healthy adults experiencing PASC who had recovered from mild COVID-19, compared to healthy control (CONT) participants without a history of COVID-19. The overall aim was to assess PASC vs. CONT group differences in the following: (1) behavioral performance and neural measures during an inhibitory Go/NoGo task and error processing and (2) spontaneous EEG measured during wakeful rest, in the context of (3) an array of self-reported variables.

## 2. Materials and Methods

### 2.1. Participants

**Figure 1:**
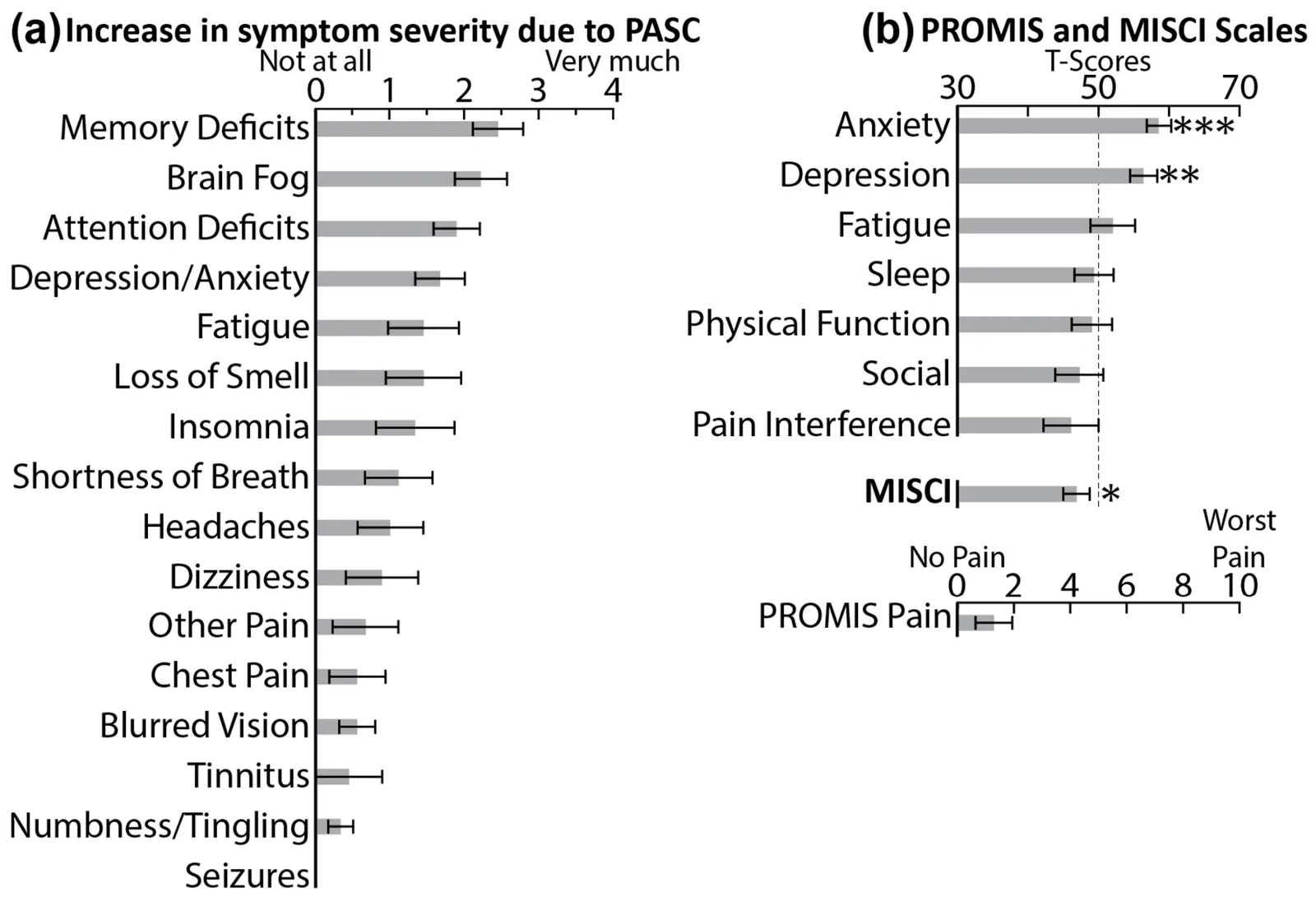
(**a**) PASC group average increase in residual symptom severity (means ± standard errors) compared to the pre-COVID-19 health status on a scale from 0 (not at all) to 4 (very much). (**b**) Average standardized T-scores (means ± standard errors) obtained from PASC participants on PROMIS (Patient-Reported Outcomes Measurement Information System) subscales [83]. They were compared to the standardized T-score mean of 50, marked with a dotted line [89]. PASC participants reported higher levels of anxiety and depression than the general normative sample. They also had lower scores on MISCI (Multidimensional Inventory of Subjective Cognitive Impairment) [86], indicating cognitive impairment. * *p* < 0.05, ** *p* < 0.01, *** *p* < 0.001.

The PASC group comprised 18 mostly young adults (M ± SD = 25.4 ± 7.1 years of age, 11 women) who had previously enjoyed good health before contracting mild COVID-19. They enrolled in the study on average 6 months (26.1 ± 16.6 weeks) after recovery, and all met the PASC definition criteria [13]. Participants were right-handed and had no history of concussions or head injuries, seizures, neurological or psychiatric diagnoses, hearing or vision problems, or other chronic health issues aside from PASC. Additionally, they reported no regular tobacco or marijuana use within the past month and illicit drug use within the past two months. Two participants reported using fluoxetine (selective serotonin reuptake inhibitor) and lisdexamfetamine, respectively, though they refrained from taking these medications at least 24 h before taking part in the study. None of the participants required hospitalization at any point. Their PASC symptom severity profile (Figure 1) was in line with other studies [5,14,21,26,80,81]. They reported that PASC symptoms had a moderate overall impact on their lives, rating it as 1.9 ± 1.2 on a scale from 0 (not at all) to 4 (very much). However, the PASC symptoms did not result in significant functional limitations in daily duties and activities (0.8 ± 1.3) on the Post-COVID-19 Functional Status Scale, with ratings ranging from 0 (no functional limitations) to 4 (severe functional limitations) [82]. Notably, participants reported higher levels of depression and anxiety compared to general population norms as determined with Patient-Reported Outcomes Measurement Information System (PROMIS) scales (Figure 1b) [83], confirming previous accounts from large-scale studies [8,10,21,80,81,84,85]. In addition, the PASC participants rated their cognitive abilities lower than the established population norms on the Multidimensional Inventory of Subjective Cognitive Impairment (MISCI) [86]. These findings are consistent with the long-term neurocognitive sequelae characterizing PASC [29,80,81,87,88].

The control group (CONT) included twenty healthy, right-handed participants (23.8 ± 3.6 years of age, 14 women) who had not contracted COVID-19. The CONT participants were recruited from the same local community as the people with PASC as part of a then-ongoing study just before the pandemic, using the same protocols and tasks. The PASC and CONT groups were matched on demographic variables, impulsivity, sensation seeking, and alcohol consumption (Table 1). However, the PASC group reported higher levels of anxiety and depression than CONT, as measured with the GAD-7 [90] and PHQ-9 questionnaires [91], as shown in Table 1. Additionally, they reported greater sleep difficulties than demographically comparable groups as assessed with the Pittsburgh Sleep Quality Index [92]. The CONT participants reported no concussions, seizures, neuropsychiatric disorders, or hearing or vision problems. The study’s procedures were approved by the local University Institutional Review Board, and all participants provided written informed consent before participating in this protocol.

### 2.2. Procedure

Prospective PASC participants underwent an intake procedure that included a screening questionnaire and an interview conducted by a trained staff member. They provided details on acute COVID-19 illness, PASC symptoms, and their impact on daily functioning [82] and quality of life. Only those prospective participants who experienced PASC symptoms for at least two months were eligible for the study [13]. PASC participants completed a comprehensive battery of assessments hosted on Qualtrics (QualtricsXM, 2020). They provided ratings of the relative increase in severity of a range of symptoms compared to their pre-COVID-19 state of health (Figure 1a). This list was based on previous studies [5,7,8,14,21,26,80,81]. The battery also included short-form PROMIS (Patient-Reported Outcomes Measurement Information System) scales [83] (Figure 1b). Raw scores from the PROMIS scales were converted to standardized T-scores, allowing for comparison with the normative sample from the U.S. population, as shown in Figure 1b [89]. Complaints of cognitive dysfunction were assessed with MISCI (Multidimensional Inventory of Subjective Cognitive Impairment, [86]) (Figure 1b). Participants reported being nearly pain-free on a scale of 0 (no pain) to 10 (worst pain imaginable). As shown in Table 1, impulsivity was evaluated with the ABIS (Abbreviated Impulsiveness Scale, [93]), and risk-taking/sensation-seeking behaviors were assessed with the BSSS (Brief Sensation Seeking Scale, [94]). Anxiety was additionally assessed with the GAD-7 (Generalized Anxiety Disorder 7-item scale, [90]) and depressive symptoms with the PHQ-9 (Patient Health Questionnaire 9-item scale, [91]). Sleep issues were assessed with the PSQI (Pittsburgh Sleep Quality Index, [92]). Participants were also asked about their habitual weekly alcohol consumption in the past six months. Upon arrival at the lab for EEG recordings, their verbal recall was assessed using a modified version of the TYM-MCI (Test Your Memory for Mild Cognitive Impairment, [95]).

### 2.3. Inhibitory Go/NoGo Task

**Figure 2:**
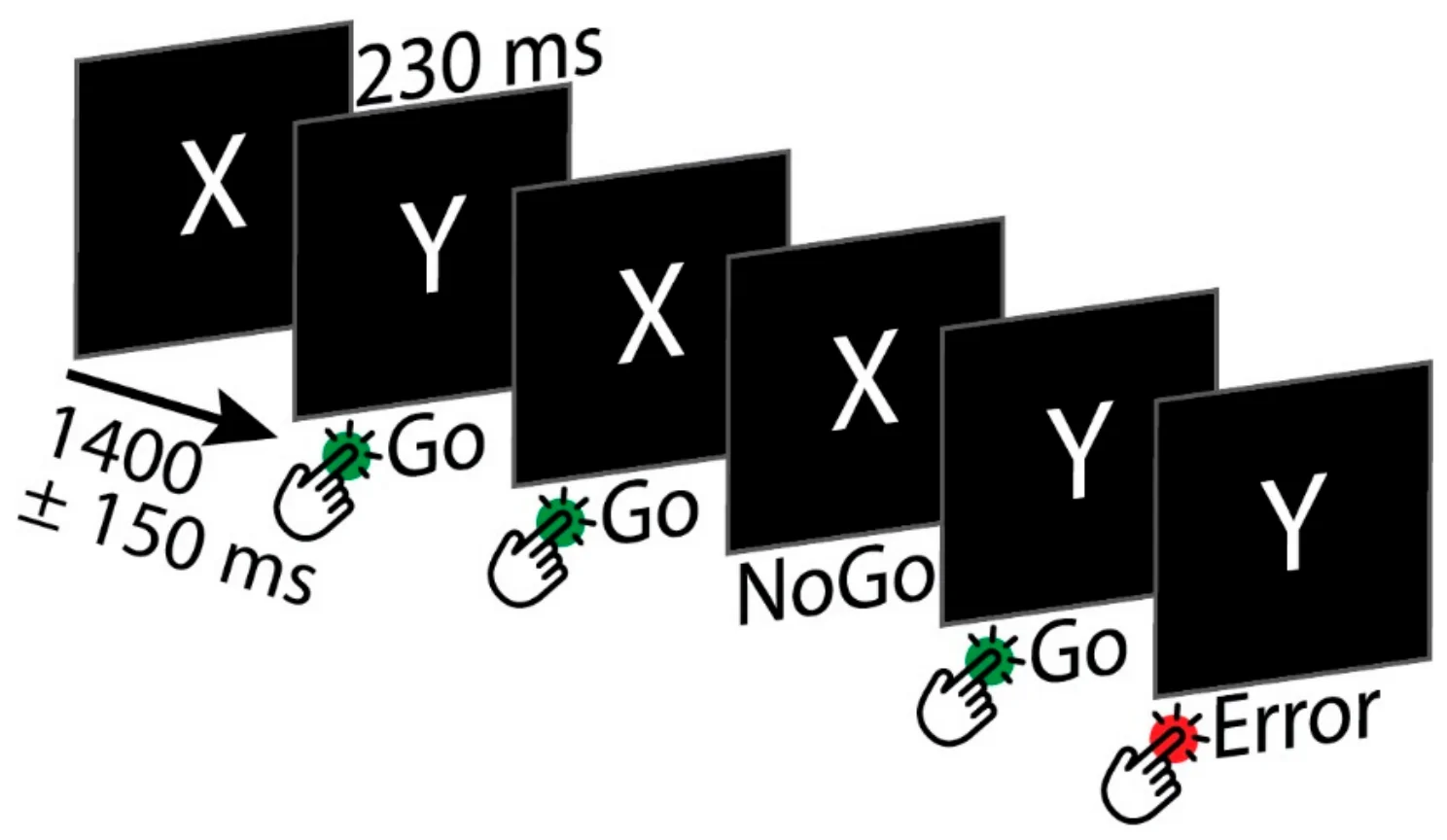
Go/NoGo task design. A pseudorandom series of “X” and “Y” letters was presented sequentially on a computer screen. Participants were instructed to press a button when the letters alternated (Go, 80% of trials) and to withhold responding to all repeated letters (NoGo, 20% of trials).

As shown in Figure 2, all participants completed an asymmetric version of the visual Go/NoGo task designed to test their ability to inhibit prepotent responses, as demonstrated in our previous studies [34,36,37,38,45,96]. A total of 685 trials were presented in white font on a black computer screen, using a stimulus-onset asynchrony (SOA) of 1400 ms ± a random jitter of 150 ms in 50 ms increments. Each letter was on screen for 230 ms, then swapped with a fixation cross until the following letter was presented. The task was conducted using the Presentation software (Neurobehavioral Systems Inc., Berkeley, USA). Responses to NoGo trials were coded as commission errors, reflecting a failure of inhibition.

### 2.4. EEG Acquisition and Analysis

Neural oscillatory activity was measured with EEG during the Go/NoGo task and a five-minute eyes-open (EO) and eyes-closed (EC) wakeful resting paradigm. EEG data were acquired using a BrainVision actiCHamp system (Brain Products GmbH, Germany) with a 500 Hz sampling rate and a 200 Hz low-pass filter. A limited electrode montage embedded in an actiCAP included Fz, Cz, C3, C4, Pz, Oz, O1, and O2. An electrode placed on the nose served as a reference, and another on the forehead served as the ground. Data were analyzed with customized MATLAB 2012a (MathWorks, Natick, MA, USA) scripts that incorporated publicly available functions from FieldTrip [97] and EEGLAB [98].

Continuous data recorded during the Go/NoGo task were bandpass filtered between 0.1 and 100 Hz, then segmented into epochs ranging from −300 ms to 800 ms relative to each stimulus onset. Response-locked epochs were obtained by segmenting data from −400 ms to 800 ms after each button-press response. To account for edge artifacts resulting from Morlet wavelet convolution, an additional 300 ms padding was added to the start and end of each epoch. The analysis of task performance identified trials with correct and incorrect responses. All epochs were downsampled to 250 Hz and inspected for artifacts utilizing specialized FieldTrip functions to visually assess metrics of variance, min/max, and z-scores for each trial. Trials with large artifacts were rejected, while eye blinks and heartbeats were identified and removed using Independent Component Analysis [99]. Data were analyzed in the time-frequency domain by convolving all epochs with Morlet wavelets within the theta band (4–7 Hz), yielding complex power spectra for each epoch in 1 Hz increments. Afterward, the padding was removed, and the spectra were averaged over the theta frequency range. The wavelet results were additionally visually inspected for artifacts. During data cleaning, 4% of trials were removed for each participant on average due to noise. The event-related theta power (er-theta) was converted to percent signal change from baseline, defined as the 300 ms preceding each stimulus-locked epoch. Response-locked epochs were also normed to the same pre-stimulus baseline period for each corresponding stimulus. This approach enabled us to evaluate group and condition differences in theta power during both correct responses and failures of inhibitory control reflected in NoGo errors. For each participant and task condition, the averaged er-theta change was quantified within a 300–400 ms window during stimulus-locked epochs to capture theta power during successful response inhibition (NoGo) and response activation (Go) across groups. Er-theta power to NoGo errors vs. correct Go responses were quantified within 0–100 ms for response-locked epochs [34,36,46,100,101]. Error processing was additionally examined by estimating co-oscillations in theta frequency over the fronto-central scalp as a function of errors. We calculated phase-locking values (PLVs) between the electrode pairs C3-C4, Fz-C4, and Fz-C3 for both an early (0–100 ms) and a later (450–600 ms) time window, with respect to erroneous and correct responses, and presented them as percent signal change relative to the pre-stimulus baseline. PLVs reflect the consistency of phase differences in the theta frequency band, providing real-time insight into long-range co-oscillations [49,50,102,103].

Continuous EEG data recorded during wakeful rest were segmented into four-second epochs and preprocessed the same way as Go/NoGo EEG data. Each epoch was likewise filtered between 0.1 and 100 Hz, downsampled to 250 Hz, and visually inspected for artifacts, aided with Independent Component Analysis [99] to detect eyeblinks and heartbeat. On average, 5% of epochs were removed due to noise. Absolute power spectra (PS) were computed within a 1–30 Hz range for each artifact-free epoch using a multi-taper Fast Fourier Transform with Hanning tapers with a frequency resolution of 0.25 Hz. Averaged PS profiles for the EO and EC conditions were calculated for each participant within the theta (3–7 Hz), alpha (8–12 Hz), and beta (15–20 Hz) frequency ranges. Power estimates were averaged across participants within each group. The alpha peak frequency (APF) was defined as the frequency with the highest power within the canonical alpha range (8–12 Hz) and was determined for each participant with an automatic algorithm. In addition to APF, the alpha power ratio was computed to aid in detecting spectral slowing. The slow-to-fast alpha ratio was calculated by dividing the power in the slow alpha band (8–10 Hz) by the power in the fast alpha band (10–12 Hz) for each participant in both conditions [104].

### 2.5. Statistical Analysis

Group differences in quantitative variables were analyzed using one-way ANOVAs, whereas categorical variables such as sex and ethnicity were evaluated using the χ2 test (Table 1). Within the PASC group, PROMIS and converted MISCI T-scores were compared to the normative population means with one-sample t-tests. Independent t-tests were used to compare PSQI scores with published data obtained from large samples with similar demographic characteristics [105,106]. On the Go/NoGo task, performance accuracy and stimulus-evoked theta were examined using a 2 × 2 mixed-model ANOVA with group (PASC, CONT) as a between-group factor and condition (Go, NoGo) as a within-subject factor. Similarly, response-locked theta and PLVs were analyzed using a 2 × 2 mixed-model ANOVA with group (PASC, CONT) as a between-group factor and response accuracy (correct Go, error NoGo) as a within-subject condition. Associations between self-reported variables, task performance, er-theta, and PLVs were determined using Pearson’s correlations. Differences in APF, alpha power ratios, and power within the theta, alpha, and beta frequency bands during the EC condition were tested using one-way ANCOVAs with group as a between-subjects factor. Scores from the GAD-7 and PHQ-9 scales were used as covariates in all analyses of neural indices to control for group differences in anxiety and depression (Table 1) and to account for their possible association with EEG measures [107]. To protect against alpha inflation in multiple comparisons, Tukey’s Honestly Significant Difference (HSD) was used to test pairwise comparisons, and corrected *p*-values are reported for these comparisons. Similarly, to control for the proportion of false positives among correlations, we used a standard False Discovery Rate (FDR) Benjamini–Hochberg procedure [108] with the FDR of 0.05. Both uncorrected (*p*) and corrected (*q*) values are reported. Of note, for a single test with a significance threshold of *p* = 0.05, the probability of a false-positive result is 5% when the null hypothesis is true. In contrast, an FDR threshold of *q* = 0.05 controls the expected proportion of false positives among a larger set of significant results at 5%. Correlations that did not survive FDR correction and do not quite reach *q* = 0.05 are reported as marginal, but not significant. Statistical analyses were performed using IBM SPSS Statistics (Version 31.0.0).

## 3. Results

### 3.1. Demographic and Self-Reported Variables

As shown in Table 1, the PASC and CONT groups were matched on demographic variables, impulsivity, sensation seeking, and alcohol intake. However, PASC participants reported higher levels of anxiety *F*(1,35) = 7.87, *p* = 0.008 and depression, *F*(1,35) = 4.49, *p* = 0.041, which was confirmed through comparisons with the PROMIS normative sample for anxiety: *t*(9) = 6.34, *p* < 0.001, and depression: *t*(9) = 4.51, *p* = 0.001 (Figure 1b). The PASC group reported higher levels of subjective cognitive dysfunction, as indicated by lower MISCI scores than the normative sample, *t*(17) = 2.59, *p* = 0.019. The PASC group additionally reported sleep difficulties, as their PSQI [92] group mean (±SD) of 9.05 ± 3.80 exceeded the threshold of 5, indicating significant sleep problems. Furthermore, their PSQI scores were much higher than those reported for demographically comparable groups, including college students, *t*(882) = 5.09, *p* < 0.0001 [105], and adults under the age of 30, *t*(23199) = 7.12, *p* < 0.00001 [106]. Taken together, higher scores on the scales measuring anxiety, depression, and sleep problems are consistent with greater neural excitability.

### 3.2. Go/NoGo Task

#### 3.2.1. Performance

**Figure 3:**
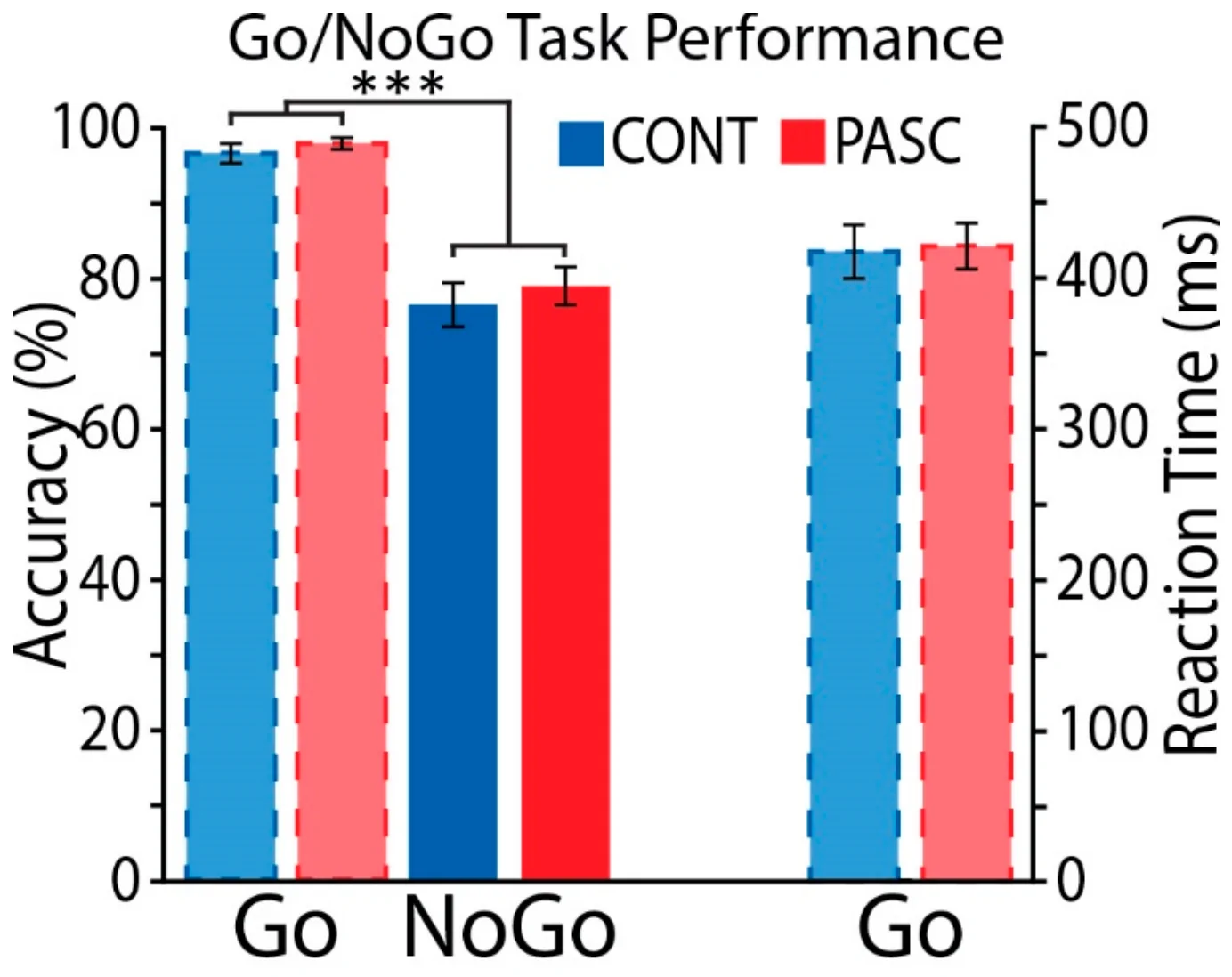
Group average response accuracy for Go and NoGo conditions, and reaction times for Go trials. As expected, accuracy was lower on NoGo than on Go trials overall, but the PASC and CONT groups did not differ in task performance. *** *p* < 0.001.

As expected, performance accuracy was lower on NoGo than on Go trials overall, *F*(1,34) = 101.44, *p* < 0.001 (Figure 3). However, there was no condition by group interaction, *F*(1,34) = 0.79, *p* = 0.381, nor an overall group difference in accuracy, *F*(1,34) = 1.39, *p* = 0.247. For the PASC group, NoGo accuracy was marginally positively correlated with memory (TYM-MCI), *r* = 0.55, *p* = 0.028, *q* = 0.059, and intelligence (WASI), *r* = 0.49, *p* = 0.044, *q* = 0.063, consistent with the contributions of the top-down executive to these functions. The PASC and CONT groups did not differ in Go reaction times, *F*(1,34) = 1.58, *p* = 0.218.

#### 3.2.2. Go/NoGo Task: Stimulus-Locked Theta on Correct Trials

**Figure 4:**
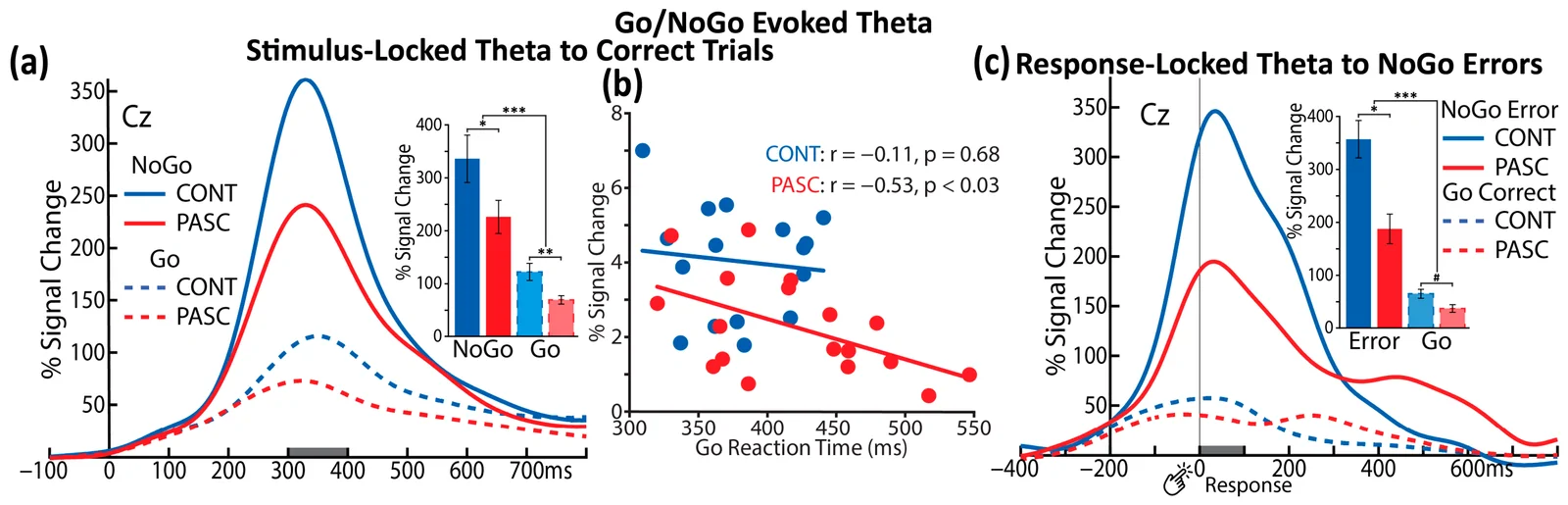
(**a**) Group-average time courses of stimulus-related theta power for correct NoGo and Go trials for the PASC and CONT groups. The histogram shows group and condition comparisons for er-theta values within the 300–400 ms window around the peak, as marked on the *x*-axis. (**b**) The scatterplot depicts the correlations between er-theta on NoGo trials and Go reaction time separately for CONT and PASC groups. (**c**) Time courses of response-locked theta during inhibitory failures (NoGo errors) and correct Go responses for both groups. The histogram displays group and condition comparisons of er-theta power within a 0–100 ms window immediately after a button press, as marked on the *x*-axis. Er-theta is expressed as a percent signal change relative to the prestimulus baseline. # *p* < 0.1, * *p* < 0.05, ** *p* < 0.01, *** *p* < 0.001.

As shown in Figure 4a, er-theta was lower in the PASC than in the CONT group for both NoGo, *F*(1,32) = 9.77, *p* = 0.014, and Go conditions, *F*(1,32) = 15.53, *p* = 0.003 (Figure 4a). As expected, NoGo trials elicited greater er-theta than Go trials overall, *F*(1,32) = 92.70, *p* < 0.001. Er-theta was associated with Go accuracy on NoGo, *r* = 0.43, *p* = 0.002, *q* = 0.012, and Go trials, *r* = −0.52, *p* = 0.001, *q* = 0.012. Furthermore, Go reaction times were negatively correlated with er-theta during NoGo, *r* = −0.49, *p* = 0.002, *q* = 0.012, and Go trials, *r* = −0.52, *p* = 0.001, *q* = 0.012 across both groups. This was especially the case for the PASC group (Figure 4b). These results suggest that better performance is associated with a more effective engagement of the motor control system during both motor execution (Go trials) and the suppression of prepotent responses (NoGo trials).

#### 3.2.3. Go/NoGo Task: Response-Locked Theta Power to NoGo Errors and Correct Go Responses

Sensitivity of theta power to the failures of inhibitory control was assessed by time-locking epochs to NoGo errors and to correct Go button presses. Response-locked theta was lower in the PASC than in the CONT group overall, *F*(1,32) = 15.79, *p* < 0.001 (Figure 4c). A group-by-condition interaction, *F*(1,32) = 10.83, *p* = 0.002, indicated that the reduction in theta power in the PASC group was especially pronounced for NoGo errors, *F*(1,32) = 14.11, *p* = 0.010, but approached significance for correct Go responses, *F*(1,32) = 6.18, *p* = 0.080. Go reaction times correlated negatively with response-locked er-theta on both error trials (*r* = −0.48, *p* = 0.003, *q* = 0.013) and Go responses (*r* = −0.54, *p* = 0.001, *q* = 0.012), suggesting that higher er-theta power is associated with more efficient processing. Additionally, er-theta to NoGo errors (Figure 4c) positively correlated with er-theta to correct NoGo trials (Figure 4a), *r* = 0.62, *p* = 0.001, *q* = 0.012, suggesting that both facets are mediated by highly related processes.

#### 3.2.4. Go/NoGo Task: Response-Related Oscillatory Synchrony, Phase-Locking Values (PLVs)

**Figure 5:**
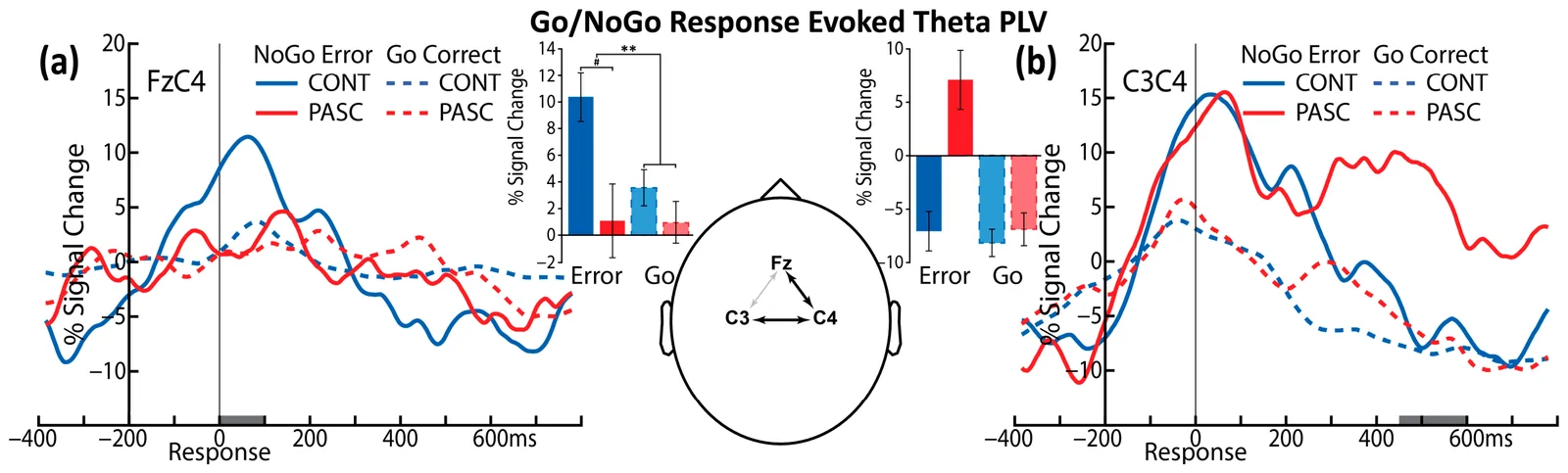
Response-related PLVs for both inhibition (NoGo) errors and correct Go responses were converted to percent signal change from prestimulus baseline. The time windows relevant to each histogram are marked on the *x*-axis. (**a**) For the Fz-C4 pair, PASC participants show marginally lower PLVs than the CONT group at the early 0–100 ms window. (**b**) However, for the C3-C4 pair, PLVs trended higher for the PASC than the CONT group at the late 450–600 ms window, which was associated with negative affect. # *p* < 0.1, ** *p* < 0.01.

Neural co-oscillatory dynamics were examined with the PLV analysis over the fronto-central scalp. At the Fz-C4 pair (Figure 5a), PASC participants exhibited marginally lower oscillatory synchrony than CONT only when failing to inhibit prepotent responses, i.e., on NoGo error trials within a 0–100 ms window, *F*(1,32) = 7.82, *p* = 0.097. NoGo errors elicited greater PLVs than Go responses overall, *F*(1,32) = 7.79, *p* = 0.009. NoGo error PLVs marginally correlated with er-theta to NoGo errors *r* = 0.34, *p* = 0.041, *q* = 0.063. Additionally, NoGo Error PLVs were marginally negatively correlated with anxiety (GAD-7) *r* = −0.33, *p* = 0.047, *q* = 0.063 and depression (PHQ-9) *r* = −0.37, *p* = 0.041, *q* = 0.063), indicating that lower engagement of inhibitory control could be associated with negative affect. At the C3-C4 pair, making an inhibitory (NoGo) error elicited greater synchrony (PLVs) than Go responses *F*(1,32) = 13.04, *p* < 0.001 during the early time window, but there was no group effect *F*(1,32) = 0.02, *p* = 0.90 or group x condition accuracy interaction *F*(1,32) = 0.01, *p* = 0.93. However, in the later (450–600 ms), post-response time window, the PLVs were trending to be greater for PASC than CONT participants to inhibitory errors F(1,32) = 4.55, *p* = 0.186. For this comparison, the uncorrected *p*-value was 0.040, but it did not survive rigorous HSD alpha correction. PLVs during Go responses were not significantly different F(1,32) = 0.08, *p* = 0.99 (Figure 5b). PLVs to NoGo errors correlated marginally with depression (PHQ-9) *r* = 0.34, *p* = 0.027, *q* = 0.059, and were marginally negatively associated with er-theta to correct NoGo trials *r* = −0.37, *p* = 0.029, *q* = 0.059.

### 3.3. Spontaneous EEG Signals During Wakeful Rest

**Figure 6:**
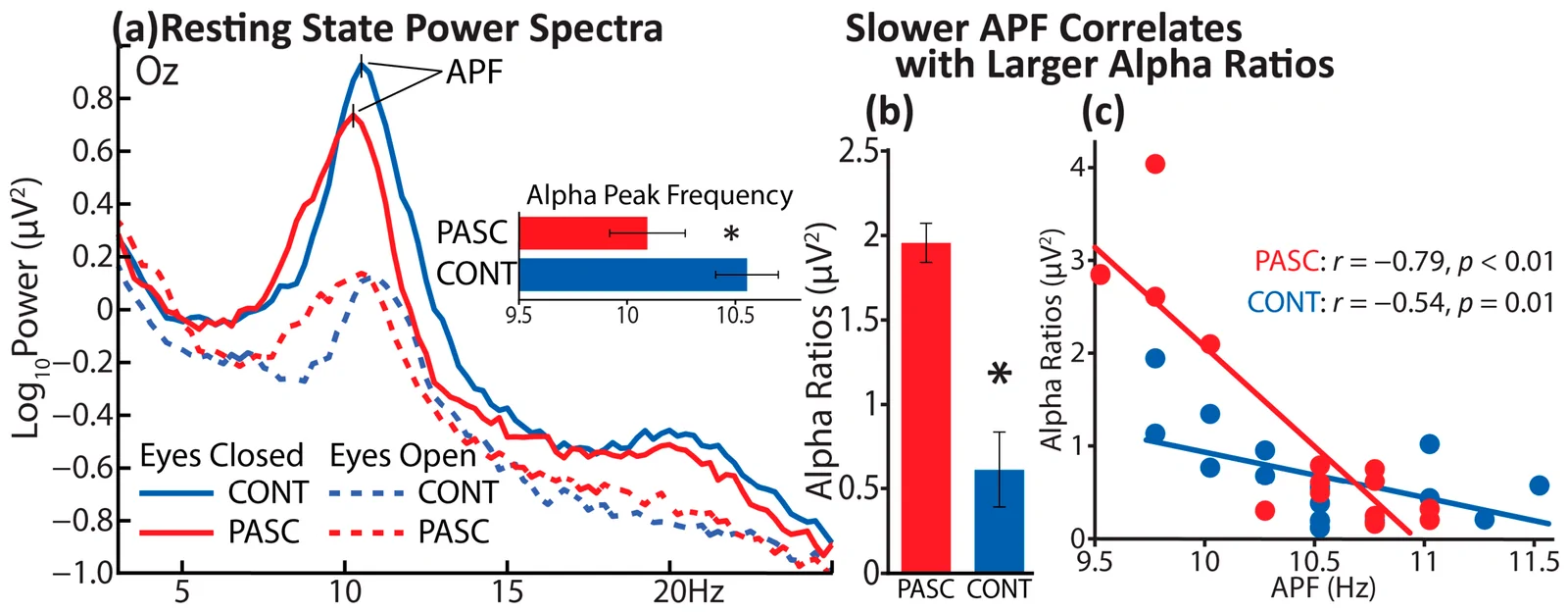
(**a**) Group-averaged power spectra were normalized to log10 values and superimposed for PASC and CONT groups across both eyes open (EO) and eyes closed (EC) conditions. The horizontal histogram shows the average alpha peak frequency, APF ± standard error for both groups in the EC condition. PASC exhibited slower APF than CONT. (**b**) The histogram depicts group-average slow-to-fast alpha ratio values ± standard error during EC. (**c**) The scatter plot shows that APF is highly correlated with alpha ratios. Correlation coefficients and *p*-values are shown separately for PASC and CONT groups. * *p* < 0.05.

The PASC group showed oscillatory slowing, as reflected in a lower APF than the CONT group (Figure 6a), based on one-way ANCOVA while controlling for anxiety and depression, *F*(1,33) = 4.59, *p* = 0.040. Oscillatory slowing in the PASC group was additionally confirmed with a main effect of group on the slow-to-fast alpha ratio (Figure 6b) after controlling for anxiety and depression. The PASC group had greater power than the CONT group in slower alpha relative to faster alpha *F*(1,33) = 6.67, *p* = 0.014 (PASC: 2.0 ± 2.4 μV2, CONT: 0.6 ± 0.5 μV2). APF and the slow-to-fast alpha ratio are negatively correlated, *r* = −0.74, *p* < 0.001, *q* < 0.001, and represent complementary facets of the same phenomenon of oscillatory slowing. Both indices of oscillatory slowing correlated with overall er-theta to correct trials and error responses. In addition, the slow-to-fast alpha ratio correlated with PLVs over the central scalp in the 450–600 ms window (Table 2). Measures of oscillatory slowing were also consistently associated with greater symptom severity.

There were no group differences in power within theta *F*(1,33) = 2.08, *p* = 0.16, alpha *F*(1,33) = 0.32, *p* = 0.58, or beta frequency ranges *F*(1,33) < 0.01, *p* = 0.95.

## 4. Discussion

To contribute to the existing literature on PASC, the current study recruited young, otherwise healthy adults who reported only a moderate overall impact on their daily lives and no significant limitations due to PASC. They were not hospitalized for COVID-19 or PASC at any point and enjoyed good health before contracting COVID-19. Notably, the PASC participants continued experiencing brain fog, fatigue, insomnia, memory/attentional deficits, heightened anxiety, depression, and other symptoms for approximately six months after recovering from COVID-19 (Figure 1 and Table 1). Overall, the clinical presentation of PASC in this cohort appears mild, but aligns with other reports [5,7,8,10,14,20,23,52,109,110,111,112,113]. Likewise, performance on the Go/NoGo task did not differ between the PASC and CONT groups, as reflected in equivalent accuracy and response speed (Figure 3). This lack of performance deficits is not surprising, given that the participants reported only mild-to-moderate PASC symptoms and no significant functional limitations in their daily activities. In contrast, other studies reported psychomotor slowing in older people with PASC (>48 years of age) who had significant levels of pre-existing and current neuropsychiatric comorbidities and hospitalization rates [114]. It is known that PASC symptoms are more severe in older individuals [115], which aligns with cognitive dysfunctions associated with hospitalization and chronic illnesses unrelated to COVID-19 [116]. To provide complementary insights, our study focused on the other side of the severity and age spectrum and studied young adults without comorbidities. While the CONT group was demographically matched and included individuals who had never contracted COVID-19, the design would have been stronger if it had included an additional control group of participants who recovered from COVID-19 without developing PASC.

Importantly, despite intact task performance, direct measures of brain activity showed weaker engagement of inhibitory control and error processing in young adults with PASC. Er-theta power was lower in the PASC group than in the CONT group (Figure 4a), with higher er-theta elicited by inhibitory NoGo trials overall. Er-theta correlated negatively with reaction times for the PASC group (Figure 4b), indicating lower engagement of inhibitory control on trials with slower response preparation and execution, consistent with previous reports [46]. Furthermore, NoGo accuracy marginally correlated with better memory and higher general intelligence, suggesting that inhibitory control could play a key role in flexible and strategic behavioral planning. Extensive evidence confirms that er-theta power is highly sensitive to decision-making, as probed by tasks that engage response inhibition and cognitive control functions [34,36,44,117,118]. The asymmetrical design of our fast-paced Go/NoGo task included infrequent and unpredictable NoGo trials, which induced occasional inhibitory failures (i.e., commission errors) on a subset of trials [33]. Error-related theta was lower in the PASC than in the CONT group (Figure 4c), indicating dysregulated cognitive control, which may contribute to difficulties with flexible behavior modification and optimizing future decisions [36,119].

It has been proposed that synchronized long-range theta co-oscillations subserve cognitive control and top-down behavioral regulation more broadly [44,49,120,121]. To examine the impact of PASC on synchronous co-oscillations, we calculated phase-locking values (PLVs) between electrodes over the fronto-central scalp. PLVs reflect the consistency of the oscillatory phase in the theta frequency band irrespective of the oscillatory amplitude [103,122]. They index the cross-cortical integrative role of theta and are sensitive to cognitive conflict [49]. PLVs measured immediately after making inhibitory errors were marginally lower in the PASC group than in the CONT group (Figure 5a), indicating atypical error monitoring. There was also a trend of weaker neural synchrony (i.e., lower PLVs) being associated with higher anxiety and depression scores. In contrast, at a later stage of post-error adjustments, error-related PLVs tended to be higher for the PASC group, which had a marginal positive association with depression. It is well established that errors are aversive and elicit negatively valenced “oh, no!” responses [33,123,124]. Thus, this observed pattern aligns with limbic engagement and negative emotions elicited by error commission. Methods with better spatial resolution estimate post-error theta activity at this approximate latency in the rostral ACC [36] and the amygdala [125], both key limbic hubs that contribute to the emotional aspect of post-error adjustments [35]. The limbic origin of exaggerated post-error activity aligns with higher levels of depression and anxiety (Table 1) as frequent PASC comorbidities [8,10,21,126]. The limbic system appears to be particularly susceptible to long-term damage following acute COVID-19 infection [127,128], as positron emission tomography (PET) has observed hypometabolism in the ACC in people with PASC [129]. Importantly, PET studies have also reported increased levels of neuroinflammatory markers in people with PASC, particularly in the ACC, striatum, and thalamus [130], which contributes to neurocognitive and affective dysregulation.

Neural dysregulation may contribute to insomnia, anxiety, and cognitive difficulties that are essential to the PASC clinical picture [7,8,131]. Other evidence has confirmed lower interhemispheric coherence and cognitive deficits in people with PASC [76,77,79,132]. Results of the current study are consistent with reports of long-lasting impairments in inhibitory control [29], as well as oscillatory asynchrony. Because cognitive functions are fundamental to well-being and daily functioning, deficits in these functions negatively affect quality of life [4,8,15,81,133].

To gain mechanistic insight into neural dysregulation, we measured EEG signals during wakeful rest [41]. The PASC group demonstrated oscillatory slowing compared to the CONT participants, as indicated by slower APF (Figure 6) and a greater slow-to-fast alpha ratio. These complementary EEG indices of a shift toward slower frequencies correlated with PASC symptoms such as attention deficits, sleep disturbances, pain, and headaches (Table 2). To account for higher scores on the screeners for anxiety and depression among the PASC participants, ratings on the GAD-7 and PHQ-9 were used as covariates in statistical comparisons. PASC-related slowing remained even after removing the influence of depression and anxiety, indicating that different mechanisms mediate the underlying neural underpinnings. Importantly, APF and slow-to-fast alpha ratio may be useful as indicators of neural dysregulation. Taken together, these findings clearly indicate that even mild PASC is associated with significant alterations in cell signaling, characterized by oscillatory slowing. Our findings align with previous reports. An early study reported slower APF and greater current density and connectivity in slower (delta) frequencies in people with PASC two months after hospital discharge, which was associated with cognitive dysfunction [76]. A larger study corroborated neural oscillatory slowing in adults, adolescents, and children who recovered from acute COVID-19 and showed increased power in the theta (4–8 Hz) band 1 to 2 months after recovery [77]. Similarly, a cohort of young, previously healthy pilots also presented greater power in the theta range after recovering from COVID-19 [134]. Furthermore, generalized slowing has also been observed, including in the theta/delta range [79], along with common PASC symptoms such as brain fog, hyposmia, and headache [76,134]. Increased power in lower frequencies is a consistent finding and an indirect measure of oscillatory power shifting, as alpha slowing increases power in the immediately adjacent theta frequencies. In our study, we observed greater power in the slower alpha band than in the faster alpha band (Figure 6b). We also measured APF directly as a prominent index of changes in dominant spectral power (Figure 6), which showed similar results. Both of these complementary indices are sensitive to EEG oscillatory features; are associated with common PASC symptoms like headaches, attention deficits, and sleep disturbances; and can serve as indices of PASC-related neural dysregulation. Furthermore, oscillatory slowing correlated with lowered er-theta to error responses and marginally with altered er-theta and late PLVs over the central scalp during the Go/NoGo task. While these associations need replication, they suggest partly common mechanisms underlying neural and cognitive dysregulation.

The nature of the mechanisms underlying these changes cannot be ascertained because the present study did not directly measure GABA/glutamate balance, inflammatory markers, or cortical excitability. However, growing research suggests that inflammation and immune activation contribute to cognitive impairments, including “brain fog” and executive deficits [9,53,67,135]. This line of evidence converges with inflammation-induced tipping of the neural balance toward neural dysregulation and hyperexcitability through extrasynaptic glutamate “spillover”, GABA downregulation, and other processes [69,70,71]. Evidence obtained with magnetic resonance spectroscopy has confirmed GABA downregulation in people with PASC, which mediated the impact of PASC on depression and was associated with insomnia [72]. Studies using transcranial magnetic stimulation (TMS) have also reported reduced cortical inhibition in people with PASC, associated with executive deficits [136,137]. Other evidence has confirmed lower interhemispheric coherence and cognitive deficits in people with PASC [76,77,79,132], possibly due to inflammatory sequelae [53,68,138] dysregulation. While more research is needed, converging evidence points to a plausible hypothesis that neural dysregulation underlies the observed EEG changes and the PASC symptomatic pattern. For instance, deficient neural inhibition underlies insomnia, anxiety, and cognitive difficulties that are essential to the PASC clinical picture [7,8,131] and are associated with increased inflammatory markers [88,139].

The present study has several limitations which should be recognized and addressed in future research. The CONT and PASC groups were equated on demographic variables, impulsivity, sensation seeking, and alcohol consumption. However, the CONT group comprised healthy individuals who had never contracted COVID-19. While this clear group difference in pandemic-related medical history is a unique feature of the study, it is also a limitation. Including an additional control group of individuals who recovered from COVID-19 without developing PASC would have helped isolate PASC-specific effects and support more nuanced conclusions. PSQI scores to assess sleep problems or TYM-MCI scores to assess memory were not available for the CONT group, limiting direct group comparisons. The study sample was also small, so results should be taken as preliminary until replicated with larger samples. Future studies should also consider supplementing neural indices with measuring power in the delta range and with direct measures of neuroinflammation and hyperexcitability to test their association and improve mechanistic interpretability.

## 5. Conclusions

This study aimed to assess the neurophysiological underpinnings of PASC in a sample of young, otherwise healthy individuals who recovered from mild COVID-19 about 6 months earlier, compared with the CONT group, which included healthy individuals who had never contracted COVID-19. The study provides valuable insights into the neural alterations associated with PASC, given the scarcity of evidence in this area. By reporting a unique range of EEG indices across two tasks and linking them with self-reported variables, this study can inform future research on PASC or related conditions. Although the groups did not differ in performance on a Go/NoGo task, young adults with PASC showed lower neural activity during both correctly inhibited and erroneous responses, confirming altered engagement of inhibitory control and error processing. In the PASC group, long-range co-oscillatory synchrony was dysregulated following error commission, which marginally correlated with negative emotion, suggesting that making errors was particularly aversive for individuals with PASC. We also detected a slowing of spontaneous oscillations, consistent with other evidence reviewed above. The oscillatory slowing correlated with common neurocognitive symptoms of PASC. Taken together, the neural indices used in the present study are highly sensitive to neurotransmission and cognitive functions, and could provide a signature of the core neural dysfunctions that characterize PASC or related syndromes.

## Figures and Tables

**Table 1:** Participant characteristics for PASC and CONT groups.

	PASC (n = 18)	CONT (n = 20)	F(1,36)/χ2	*p*
% Women	61%	70%	0.33 ^a^	0.564
% White/Non-Hispanic	83%	70%	5.14 ^a^	0.076
Age	25.44 ± 7.14	23.75 ± 3.63	0.88	0.355
Impulsivity (ABIS)				
Attention	1.74 ± 0.30	1.83 ± 0.45	0.35	0.558
Motor	1.79 ± 0.34	1.73 ± 0.41	0.21	0.651
Non-Planning	1.89 ± 0.55	1.87 ± 0.48	0.01	0.910
Sensation seeking (BSSS)	25.67 ± 5.70	25.95 ± 5.23	0.02	0.898
Anxiety (GAD-7)	4.56 ± 3.17	2.00 ± 2.33	7.87	**0.008**
Depression (PHQ-9)	4.61 ± 3.62	2.53 ± 2.25	4.49	**0.041**
Avg Drinks/Week	3.29 ± 3.70	2.65 ± 1.89	0.46	0.503
Sleep Quality Total (PSQI)	9.00 ± 4.20			
Memory (TYM-MCI)	9.50 ± 3.56			

Group means ± standard deviations were calculated for all quantitative variables, and group differences were determined with one-way ANOVAs. Categorical variables (sex and ethnicity) are presented as percentages, and χ2 tests evaluated group differences, marked with ^a^. Statistically significant *p*-values are marked in bold. ABIS: Abbreviated Impulsiveness Scale, BSSS: Brief Sensation Seeking Scale, GAD-7: Generalized Anxiety Disorder, PHQ-9: Patient Health Questionnaire (depression), PSQI: Pittsburgh Sleep Quality Index, TYM-MCI: Test Your Memory for Mild Cognitive Impairment.

**Table 2:** Original and FDR-adjusted correlations between APF, alpha ratios, and self-reported variables.

	APF				Alpha Ratios	
	*r*	*p*	*q*	*r*	*p*	*q*
APF				**−0.74**	**<0.001**	**<0.001**
Alpha Ratio	**−0.74**	**<0.001**	**<0.001**			
Correct Er-Theta Avg	0.29	0.124	0.129	−0.37	0.040	0.063
Error er-Theta	0.37	0.031	0.057	**−0.41**	**0.016**	**0.044**
PLV (C3-C4)	−0.23	0.196	0.205	0.35	0.040	0.063
Headaches	**−0.62**	**0.006**	**0.022**	0.44	0.065	0.085
Memory Deficits	−0.59	0.074	0.094	0.68	0.032	0.059
Attention Deficits	−0.56	0.093	0.108	**0.74**	**0.015**	**0.046**
Brain Fog	−0.44	0.097	0.110	0.55	0.033	0.059
Insomnia	−0.42	0.082	0.098	0.25	0.309	0.309
Fatigue	−0.40	0.101	0.111	0.42	0.081	0.098
Sleep Disturbances (PSQI)	**−0.86**	**0.001**	**0.012**	0.64	0.046	0.063
Pain Interference (PROMIS)	**−0.83**	**0.003**	**0.013**	**0.84**	**0.002**	**0.012**
Pain (PROMIS)	**−0.77**	**0.018**	**0.048**	**0.72**	**0.018**	**0.048**
Motor Impulsiveness (ABIS)	−0.40	0.023	0.055	0.37	0.032	0.063

Pearson’s correlations were carried out between the two EEG measures of oscillatory slowing (alpha peak frequency (APF) and alpha ratios), neurocognitive PASC symptoms, and other self-reported variables within the PASC group. APF is the frequency with the greatest power within the alpha (8–12 Hz) range. Alpha ratio is defined as slow (8–10 Hz) alpha power divided by fast (10–12 Hz) alpha power. An oscillatory shift toward slower frequencies is reflected in lower APF and a higher alpha ratio. Headaches, memory and attention deficits, brain fog, insomnia, pain, and fatigue are frequently reported symptoms of PASC (Figure 1a). PSQI: Pittsburgh Sleep Quality Index, ABIS: Abbreviated Impulsiveness Scale, PROMIS: Patient-Reported Outcomes Measurement Information System. Uncorrected *p*-values are reported as *p* while the Benjamini–Hochberg [108] corrected *p*-values are reported as *q*. All bolded values are significant after rigorous FDR correction with a threshold of 0.05.

## Data Availability

Research data are available upon a reasonable request. Please contact Ksenija Marinkovic at kmarinkovic@sdsu.edu.

## Abbreviations

The following abbreviations are used in this manuscript:

PASC	Post-Acute Sequelae of SARS-CoV-2 infection
CONT	Control group
EEG	Electroencephalography
APF	Alpha Peak Frequency
PLV	Phase-Locking Values
EC, EO	Eyes closed, eyes open conditions during wakeful rest
PS	Power spectra
